# Environment and Co-occurring Native Mussel Species, but Not Host Genetics, Impact the Microbiome of a Freshwater Invasive Species (*Corbicula fluminea*)

**DOI:** 10.3389/fmicb.2022.800061

**Published:** 2022-04-04

**Authors:** Marlène Chiarello, Jamie R. Bucholz, Mark McCauley, Stephanie N. Vaughn, Garrett W. Hopper, Irene Sánchez González, Carla L. Atkinson, Jeffrey D. Lozier, Colin R. Jackson

**Affiliations:** ^1^Department of Biology, University of Mississippi, Oxford, MS, United States; ^2^Department of Biological Sciences, University of Alabama, Tuscaloosa, AL, United States

**Keywords:** freshwater biodiversity, invasive species microbiome, microbial source tracking, RADseq, species interactions, Unionidae

## Abstract

The Asian clam *Corbicula fluminea* (Family: Cyneridae) has aggressively invaded freshwater habitats worldwide, resulting in dramatic ecological changes and declines of native bivalves such as freshwater mussels (Family: Unionidae), one of the most imperiled faunal groups. Despite increases in our knowledge of invasive *C. fluminea* biology, little is known of how intrinsic and extrinsic factors, including co-occurring native species, influence its microbiome. We investigated the gut bacterial microbiome across genetically differentiated populations of *C. fluminea* in the Tennessee and Mobile River Basins in the Southeastern United States and compared them to those of six co-occurring species of native freshwater mussels. The gut microbiome of *C. fluminea* was diverse, differed with environmental conditions and varied spatially among rivers, but was unrelated to host genetic variation. Microbial source tracking suggested that the gut microbiome of *C. fluminea* may be influenced by the presence of co-occurring native mussels. Inferred functions from 16S rRNA gene data using PICRUST2 predicted a high prevalence and diversity of degradation functions in the *C. fluminea* microbiome, especially the degradation of carbohydrates and aromatic compounds. Such modularity and functional diversity of the microbiome of *C. fluminea* may be an asset, allowing to acclimate to an extensive range of nutritional sources in invaded habitats, which could play a vital role in its invasive success.

## Introduction

The introduction of species outside of their native range through human activities is an accelerating phenomenon worldwide (Seebens et al., 2017). Invasive species, spreading aggressively after introduction, have important negative ecological consequences on the ecosystem they invade and are the second leading cause of species endangerment and extinction (Bellard et al., 2016). As with other organisms, invasive species live and interact with a diverse community of microorganisms, their microbiome, which is an integral part of their biology and ecology (Bahrndorff et al., 2016). While the composition and function of microbiomes have been examined for many host species, they have rarely been assessed in an invasion context (but see, e.g., Goddard-Dwyer et al., 2021).

The influence of the microbiome on invasive species success, while increasingly recognized in plants (Kowalski et al., 2015), is still largely unknown in animals (Bahrndorff et al., 2016). This is striking given that numerous hypotheses for the success or failure of the invasion process involve mutualistic or antagonistic partners of invasive and native species, which can include microbes (Lockwood et al., 2013a,b). It has been suggested that a higher variability or diversity of functions displayed by the gut microbiome may benefit a host’s capacity to adapt to a more extensive range of nutritional niches, therefore facilitating its establishment in invaded areas (Bahrndorff et al., 2016; Goddard-Dwyer et al., 2021). The microorganisms transported by an invasive species may also pose a risk to native populations through the transmission of pathogens (Lymbery et al., 2014), or through the modification of environmental microbial communities that may be less beneficial to the native hosts (Coats and Rumpho, 2014).

As with any organism, microbiomes associated with invasive species can be driven by both host-specific (e.g., host condition, life history, genetics) and environmental factors (e.g., water physichochemistry, diet) (Chiarello et al., 2019). Additionally, while invasive species may carry microorganisms that are co-introduced from their native range, they may also lose much of their microbiome during an invasion and thus may acquire novel microbes locally (Parker et al., 2006). This makes the invasion history, such as the number of distinct introductions and subsequent patterns of spatial expansion, important when assessing the microbiome diversity of invasive species (Parker et al., 2020). Interactions with local native communities may be particularly important when the invasive species occupies the same functional role as co-occurring native fauna, as similar niche requirements should increase encounters between native and invasive species, therefore increasing the potential for microbe acquisition (Parker et al., 2006; Shelby et al., 2016). Thus, investigating the microbiome of established invasive populations across ecological gradients provides an opportunity to understand the relative influence of host-specific and environmental influences in shaping the microbiome of invasive species during the invasion process.

Invasive species in freshwater habitats are of particular concern, as these ecosystems are among the most diverse and vulnerable on the planet and have exhibited the most dramatic decreases in biological diversity since the 1970s (Darwall et al., 2018; Lopes-Lima et al., 2018; Albert et al., 2021). Asian clams in the genus *Corbicula* are among the most problematic invasive freshwater species, having spread rapidly from their native range in Eastern Asia, Australia, and Eastern Africa, to a worldwide distribution in just a few decades (Lee et al., 2005; Sousa et al., 2008). The exact number of species within the genus *Corbicula* is still unclear, but most invasive lineages are classified as *Corbicula fluminea* or *fluminalis* (Sousa et al., 2008). The United States populations are referred to as *Corbicula fluminea* (National Invasive Species Information Center^1^, February 2021), but belong to five genetically distinct morphotypes (Haponski and Foighil, 2019). Corbicula fluminea reproduces androgenetically, where the nuclear DNA of juveniles is derived entirely from the male parent (Ishibashi et al., 2003), and clonal reproduction of introduced lineages has contributed to their rapid expansions (Lee et al., 2005; Haponski and Foighil, 2019).

*Corbicula fluminea* invasions are especially concerning because of their potential impact on native bivalve populations, particularly mussels in the family Unionidae (Sousa et al., 2008; Haag, 2019). Freshwater mussels play important roles in ecosystems through their filter-feeding (Vaughn and Hoellein, 2018) and are among the most threatened faunal groups worldwide, with 45% of the described species being threatened, endangered, or extinct (Lopes-Lima et al., 2014a,^2018^). Freshwater mussels are typically slow growing and long-lived (most living ∼6-50 years, Haag, 2012) and often occur in dense multi-species aggregations, yet both their diversity and abundance have been declining dramatically (Haag, 2019). *C. fluminea* occupies the same functional guild as unionids (i.e., filter-feeding bivalves) but shows a faster growth rate and shorter life span (1-5 years) that, together with earlier sexual maturity and clonal reproduction, allows it to reproduce and disperse more rapidly than native mussels and to recover more quickly following perturbations (McMahon, 2002; Sousa et al., 2008; Ferreira-Rodríguez et al., 2018b). Increasing densities of *C. fluminea* may thus replace unionid populations in the wild (McMahon, 2002; Ferreira-Rodríguez et al., 2018b) and pose a risk to remaining populations (Sousa et al., 2008; Haag, 2019). While field assessments in Western Europe and North America document variable spatial overlap between invasive *C. fluminea* and native mussel populations (Vaughn and Spooner, 2006; Ferreira-Rodríguez et al., 2018a), experiments have revealed the negative impact of *C. fluminea* on mussel growth and physiology, with the most likely cause being competition for space and/or resources, as *C. fluminea* typically displays higher individual filtration rates than native mussels (Ferreira-Rodríguez et al., 2018a,b; Haag, 2019).

Despite these possible threats to native mussels, the microbiome of *C. fluminea* and its relationship to native mussel microbiomes have never been examined. The unionid microbiome has been increasingly studied over the past few years, suggesting some degree of species-specificity, where mussel species collected from the same environment have distinct microbiomes (Weingarten et al., 2019; Liu et al., 2020; McCauley et al., 2021). However, while opportunistic bacteria are detected within moribund mussels or during mass mortality events, little is known about potential bacterial pathogens of mussel populations in the wild (Grizzle and Brunner, 2009; Leis et al., 2019; McElwain, 2019; Richard et al., 2021), or the risk of a transmission of such pathogens from exotic species such as *C. fluminea*. Whether *C. fluminea* likewise harbors its own distinctive microbiome, influences the microbiome of native freshwater mussels, or has acquired a microbial community that reflects that of the local environment and/or one of the native mussels remains unknown. Further, the unique invasion history and reproductive mode of *C. fluminea* clonal lineages may influence microbiome diversity and transmission within and among populations. Thus, understanding the distribution of different clonal lineages or fine-scale population genetic structure could provide additional clues into the respective roles of host-specific and environmental influences. Given the potential negative impacts on native mussel communities, investigating the diverse factors that may contribute to *C. fluminea* microbiome diversity alongside native mussel microbiome will help better elucidate possible impacts on freshwater mussel communities.

**FIGURE 1:**
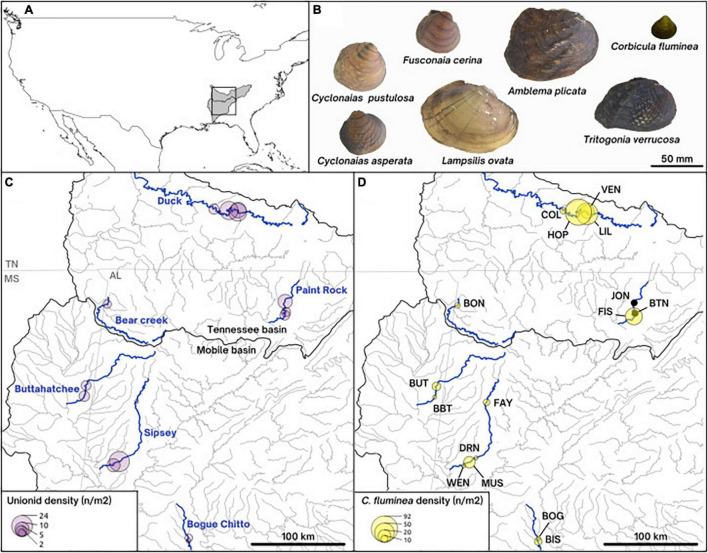
Map of the collecting sites **(A,C,D)** and pictures of the shells **(B)** of the species studied. **(A)** Overall map of the United States of America, showing the Mobile and the Tennessee river basins shaded in gray. **(C)** Density of unionids on our collecting sites along the six rivers of study. **(D)** Density of the invasive Asian clam *Corbicula fluminea* on collecting sites and site names represented by three letters. **(B)** On-scale pictures of representative shells of the native unionid species included in this paper (*Lampsilis ovata*, *Cyclonaias pustulosa*, *Cyclonaias asperata*, *Fusconaia cerina*, *Tritogonia verrucosa*, and *Amblema plicata*) and the invasive *Corbicula fluminea*.

We report the first investigation into the microbiome of *C. fluminea* populations that co-occur along a gradient of native freshwater mussel assemblage density in the Mobile and Tennessee River Basins (Figure 1; Hopper et al., 2021). We describe the diversity and inferred function of the microbiome of *C. fluminea* and assess the main differences compared to that of co-occurring native mussels. By integrating population genomic data, we examine the genetic structure of *C. fluminea* in the Mobile and Tennessee Basins and test whether ancestry contributes to microbiome diversity, either from clonal lineage or within-lineage genetic variation. Lastly, we examine if the presence and/or higher densities of *C. fluminea* may lead to changes in the microbiome of co-occurring native mussels and assess the potential reciprocal influence of the native mussel microbiome on that of *C. fluminea*. By examining how spatial-environmental variation, population genetic structure, and co-occurring mussel communities interact to influence microbiome composition, we provide insights into host-specific and environmental factors driving the microbiome of established invasive freshwater species.

## Materials and Methods

### Study Area

Samples were collected from six rivers in the Mobile and Tennessee River Basins, in the southeastern United States (Figure 1). The southeastern United States is a hotspot for freshwater mussel biodiversity, containing ∼90% of the North American diversity (Parmalee and Bogan, 1998; Williams et al., 2008). This extraordinarily diverse region has been severely degraded by anthropogenic activity, such that 95% of the 70 United States federally protected mussel species occur in this region (Williams et al., 2008). Although *C. fluminea* has been considered established in the southeastern United States for more than 50 years (US Geological Survey, 2021), complete accounts of *C. fluminea* invasion timing and quantitative population estimates are rarely available where mussels are found (Benson and Williams, 2021). On our 16 study sites (1-4 per river), *C. fluminea* density ranged from low to very high (averaging 0.5-92 individuals/m^2^) and was generally correlated to native mussel densities, which ranged from 0.6 to 23 individuals/m^2^ (as reported by Hopper et al., 2021; Figure 1).

### Specimen Collection and Environmental Measures

We collected 180 specimens of *C. fluminea* and 144 specimens of Unionidae belonging to six species (*Lampsilis ovata*, *Cyclonaias pustulosa*, *Cyclonaias asperata*, *Fusconaia cerina*, *Tritogonia verrucosa*, and *Amblema plicata*) between July and September 2019 (Figure 1 and S1-Supplementary Table 1). We collected 3-28 individuals of *C. fluminea* per site, along with up to 10 individuals each from up to three native mussel species. Mussel species were identified morphologically by author C.L.A.

All specimens were placed on ice and transported back to the University of Alabama on the same day of collection, where they were flash-frozen and stored at −80°C. The entire gastrointestinal tracts of mussels and *C. fluminea* were subsequently excised using sterile dissecting equipment and transported on dry ice to the University of Mississippi for microbiome analysis. For population genomic analysis, *C. fluminea* mantle tissue was clipped and stored in molecular biology grade absolute ethanol at −80°C.

Three surface sediment and three 120-mL water samples were collected from each site at the time of sampling. Water samples were filtered through sterile 1 μm pore size glassfiber filters (25 mm diameter; Millipore) placed in sterile tubes. Water temperature, pH, conductivity, and dissolved oxygen were measured at each site using aYSI DO Probe (YSI Inc., Yellow Springs, OH, United States), and 50 mL water samples were collected to determine concentrations of dissolved organic carbon (DOC), Soluble Reactive Phosphorus (SRP), soluble ammonium (NH_4_+), soluble nitrite (NO_2_−), soluble nitrate (NO_3_−). Sediment granulometry was also determined for each sample site. Values of these parameters and methodology are described in S1-Supplementary Table 2.

### Microbial DNA Extraction, 16S rRNA Gene Sequencing, and Sequence Processing

A subset of 3-8 *C. fluminea* specimens per site was selected for microbiome analysis for a total sample size of 80 *C. fluminea* and 144 native mussels (S1-Supplementary Table 2). Bivalve gut tissue was ground using sterile pellet pestles with the extraction buffer from a PowerSoil Pro kit (Qiagen, Germantown, MD), and bacterial DNA was extracted as described previously (McCauley et al., 2021). DNA from sediment and filtered water (seston) samples were extracted following PowerSoil Pro kit instructions. Dual-indexed barcoded primers were used to amplify the V4 region of the 16S rRNA gene of the extracted DNA from each sample following established techniques (Kozich et al., 2013; McCauley et al., 2021). This hypervariable region was chosen following previous work on unionid microbiome using Illumina MiSeq technology (Weingarten et al., 2019; Aceves et al., 2020; Richard et al., 2021), and recommendations from the Earth Microbiome Project^2^ (01/2022). The amplified 16S rRNA gene fragments were combined and spiked with 20% PhiX before being sequenced on an Illumina MiSeq at the University of Mississippi Medical Center Molecular and Genomics Core Facility.

Raw sequences were processed using the DADA2 R-package in R version 3.6.3 (R Core Team, 2020b). We followed the general methodology available on the DADA2 Github^3^ (11/2020). We filtered sequences with more than two and five estimated errors on forward and reverse reads, respectively, and truncated reads on their 3′ end at the first base where quality dropped under a quality score of 2 (TrunQ = 2). After estimation of error rates, Amplified Sequence Variants (ASVs) were predicted and merged using default parameters. Chimeras were removed using the consensus method in “removeBimeraDenovo” function. Any final ASV out of a range of 243-263 base pairs was then removed, leaving a total of 57,556 ASVs in the un-rarefied dataset of 318 samples. Bivalve microbiomes contained 4,042 to 196,506 sequences. To ensure comparable alpha- and beta-diversities, we randomly rarefied bivalve samples to 4,000 sequences. Environmental samples (sediment, seston) contained fewer sequences (2,374 - 16,587 and 2,100 - 34,963, respectively), and were rarefied to 2,000 sequences per sample. After rarefaction, 31,091 ASVs remained in the entire dataset containing all bivalves and environmental samples. Coverage was assessed by Chao’s non-parametric indicator using the “entropart” R-package (Marcon and Hérault, 2015) and averaged 0.98 ± 0.02 (Mean ± Standard Deviation, here and elsewhere) across samples after rarefaction.

### Microbial Phylogeny and Diversity Indices

Alpha-diversity (Shannon alpha-diversity) was assessed using the ‘vegan’ R-package and expressed in an equivalent number of species (Dixon, 2003; Jost, 2007). To compute phylogenetic diversity, ASV sequences were incorporated into the GreenGenes 99% phylogenetic tree version 13.8 (McDonald et al., 2012) using SEPP software (Janssen et al., 2018) implemented in QIIME2 (Bolyen et al., 2019), using default parameters. Phylogenetic richness, based on the phylogenetic distance between ASVs, and phylogenetic diversity, taking into account both phylogenetic distances and ASV relative abundance, were, respectively, assessed using Faith’s PD and the index of Allen using “picante” (Kembel et al., 2010) and “entropart” R-packages. Phylogenetic beta-diversity was assessed using the weighted (W-) and unweighted (U-) versions of Unifrac on microbial phylogeny using GUniFrac R-package (Chen, 2021).

### Statistical Analysis of Phylogenetic and Taxonomic Data

All data visualizations were made using the “ggplot2” R-package (Wickham, 2016). Comparison of alpha-diversity between *C. fluminea* and native mussels was assessed using Wilcoxon signed-rank tests, using all native mussel species at once, and separately for each mussel species. Rarefaction curves for each sample were obtained using “vegan” and averaged per species, before plotting. To assess differences in the overall structure of the gut microbiome, U- and W-Unifrac dissimilarities were plotted along the first two axes of a Principal Coordinates Analysis (PCoA) ordination, and significant differences between species, sites, and rivers were assessed using PERMANOVAs. *Post hoc* pairwise comparisons of microbiome structure were assessed using ‘‘pairewiseAdonis^4^.” Correlation between microbiome dissimilarities and physicochemical characteristics on site were assessed using the envfit function in “vegan,” removing sites for which we didn’t have all measurements (S1-Supplementary Table 2). *Tritogonia verrucosa* specimens presented the highest variability and made the ordinations difficult to interpret. Therefore, to ease visualizations, we performed PCoAs without *T. verrucosa*. Separate PCoAs, including *T. verrucosa*, are provided in Supplementary Information S2. To assess the geographical variation within *C. fluminea* microbiome, Spearman correlation tests were performed on U- and W-Unifrac dissimilarities and geographical distances between sites along the same river.

Individual LefSe analyses were computed to identify which microbial taxa differed between *C. fluminea* and each mussel species consistently across all sites (Segata et al., 2011). Co-variation of mussel and co-occurring *C. fluminea* gut microbiomes were assessed using 500 Mantel tests computed in “vegan,” on random subsamples of the same number of *C. fluminea* and native mussel specimens on each site. The median of the 500 *p*-values was computed and the distribution of Spearman’s correlation values was visualized using boxplots. The potential contribution of the microbiome of *C. fluminea* to that of native mussels was assessed using FEAST (Shenhav et al., 2019) with each native mussel specimen as a “sink” and all other co-occurring microbial communities (microbiomes of other mussels, *C. fluminea*, seston, and sediment) as “sources.” Conversely, the potential influence of the microbiome of native mussels over co-occurring *C. fluminea* was assessed using each native mussel specimen as a “source” and *C. fluminea* as a “sink.” Such reciprocal influence was then compared across recipient or source species (i.e., distinct mussel species and *C. fluminea*) and sites using separated Kruskal-Wallis tests (KW). They were then correlated with physicochemical and biotic variables (native mussel and *C. fluminea* density, native mussel richness) using separated Pearson’s correlation tests on scaled data using the “psych” R-package (Revelle, 2021).

### Microbial Functional Inferences and Functional Diversity

Functional inferences from the ASV community table and ASV sequences were obtained using PICRUST2 using default parameters except for a similarity cutoff of 0.75 to remove poorly aligned sequences (Douglas et al., 2020). The inferred enzymatic functions were aggregated into metabolic pathways according to MetaCyc database release May 2020, using the same software. Predictive abundances of pathways were transformed using a *clr* transformation of predicted expression levels using the “microbiome” R-package (Lahti et al., 2019), and samples were visualized using a Principal Component Analysis (PCA) in the “phyloseq” R-package (McMurdie and Holmes, 2013). Functional Bray-Curtis dissimilarities were computed on the transformed functional table, using “vegan” in order to test the overall differences in predicted functions from mussel vs. *C. fluminea* microbiome, using PERMANOVAs in “vegan.” To identify which pathways were significantly enriched in *C. fluminea* compared to native mussels, we performed a DESeq2 analysis with a negative binomial generalized linear model (*P* < 0.05) using “phyloseq” and “DESeq2” R-packages (Love et al., 2014). The parent class for each significant pathway was manually recorded from the MetaCyc database to simplify result visualization.

Potential structural diversity of degraded compounds was assessed by recording the Simplified Molecular Input Line-Entry System (SMILES) code of each entry compound of all pathways belonging to the Degradation-Utilization-Assimilation class. Pairwise structural dissimilarities between every compound were computed using RDKit (Landrum, 2021), and averaged for each degradation pathway. The obtained dissimilarities were then used to reconstruct a dendrogram using the ‘stats’ R-package, which was used as an input in GUniFrac to assess the predicted structural richness (unweighted Unifrac) of potentially degraded compounds (hereafter, “degradation potential”).

### Population Genomics of *Corbicula fluminea*

#### Restriction Site-Associated DNA Library Construction and Sequencing

To evaluate the population structure of *C. fluminea*, we used the Best-RAD protocol (Ali et al., 2016) for reduced representation genomic sequencing (Ali et al., 2016; Andrews et al., 2016) of 185 *C. fluminea* from the sites described above (Figure 1 and S1-Supplementary Table 1). DNA was isolated from mantle clips using Qiagen DNeasy kits (Germantown, ML, United States). DNA was normalized to 10 ng/μl and digested with the restriction enzyme *Sbf*I-HF (New England Biolabs, Ipswich, MA, United States), followed by ligation of oligonucleotides containing unique 8 base pair Hamming barcodes for each individual. Barcoded samples were combined into two sample pools (94 and 91 haphazardly distributed samples each) and sonicated using a Covaris M220 focused ultrasonicator (COVARIS, INC, Woburn, MA, United States) to generate fragments with a mean of 550 bp. RAD-tag fragments for each sample pool were isolated with streptavidin beads, and biotinylated groups were removed by Sbf-1 digestion. A NEBNext Ultra™ kit (New England Biolabs, Ipswich, MA) was used to prepare Illumina sequencing libraries with unique dual index primers for each sample pool, using 12 PCR cycles to amplify the RAD-tags. Samples were sequenced on an Illumina HiSeq X (Illumina Inc., San Diego, CA, United States) to produce 150 bp paired-end reads (Psomagen, Rockville, ML, United States).

#### Bioinformatics

Illumina reads were demultiplexed and quality filtered using Stacks v2.53 (Catchen et al., 2013) process_radtags (parameters -c, -q, -r, –best-rad, others default). 173 samples (> 500,000 reads per sample) were retained for downstream analyses. Reads were mapped to the 18 major linkage groups in the chromosome-level *C. fluminea* genome assembly (Zhang et al., 2021) using BWA-mem v (Li and Durbin, 2009). Alignments were sorted with samtools v1.10 (Li et al., 2009), and duplicate reads were removed with picard v2.18.9 MarkDuplicates^5^. Single Nucleotide Polymorphisms (SNPs) were called using freebayes v1.2.0 (Garrison and Marth, 2012), with the minimum coverage across samples set at 100 and including monomorphic loci (–report-monomorphic). All data processing of variant call files (vcf) used VCFtools v0.1.16 (Danecek et al., 2011) and statistical analyses using R version 4.0.3 (R Core Team, 2020a). We first removed indels and retained sites with a maximum of two alleles, a minimum sequence coverage of five, and a maximum of 10% missing data. This vcf was used to calculate nucleotide diversity (π) for each site, including monomorphic sites, using –site-pi in vcftools; π was averaged for each chromosome for individuals in each river. After calculating π, for other population genetic analyses, the data set was filtered to only include variant sites (–min-alleles 2) with a minimum quality threshold of 10 (–minQ 10) and a minor allele frequency of 5% (–maf 0.05). SNPs with a minor allele frequency < 0.05 were removed to reduce the impact of low frequency alleles and possible genotyping error (Rochette et al., 2019). Given that *C. fluminea* is clonal and based on other population genomic studies of this species (Haponski and Foighil, 2019), it was anticipated that there would be substantial excesses in observed heterozygosity (Balloux et al., 2003; Stoeckel et al., 2006). The inbreeding coefficient FIS was thus calculated in “hierfstat” R-package v0.5-7 using the function boot. Pfis with 1,000 bootstraps (Goudet and Jombart, 2021). After observing strongly negative FIS values (see Results), an inspection of the data revealed many SNPs that were fully heterozygous in all samples. We elected to remove SNPs with fixed observed heterozygosity prior to population structure analyses and then thinned loci to ≤ 1 SNP per kb to retain no more than one SNP per RAD-tag locus. This final dataset of 5,225 SNPs was used for all population structure and genetic distance estimations below.

The R-package “vcfR” v1.12.0 was used for import and data format conversion (Knaus and Grünwald, 2017). Individual-level differentiation was examined using a dissimilarity matrix. The “poppr” v2.9.2 package function diss.dist was used to create a distance matrix of the percent allelic differences between individuals (Kamvar et al., 2014, 2015). Population structure was visualized using discriminant analysis of principal components (DAPC) performed in “adegenet” v2.1.4 (Jombart, 2008) and plotted using “ggplot2.” To examine the genetic differentiation among populations, pairwise FST was calculated (significance tested by 1,000 bootstraps) in “StAMPP” v1.6.2 (Pembleton et al., 2013). To assess the geographical patterns within *C. fluminea* genetics, Spearman tests were performed on individual genetic dissimilarities and river distances between sites along the same river. The overall correlation between individual genetic variation and microbiome was then computed using Mantel tests performed on the 73 individuals in common the microbiome and RADSeq datasets using “vegan” (999 permutations), performed both globally and within each river. A population-level Mantel test was also performed to assess a possible correlation between pairwise FST and U- and W-Unifrac distances computed for site-averaged microbiomes (i.e., averaged ASV relative abundances for all *C. fluminea* from the same site).

#### DNA Barcoding

To confirm the clonal lineage assignment of individuals included in the analysis, a subset of individuals from each location (*N* = 47 total) were amplified for the mitochondrial cytochrome c oxidase subunit I (COI) gene using the LCO1490 and HCO2198 (Folmer et al., 1994) DNA barcoding primers. PCR and sequencing (in both primer directions) following methods in Lozier et al. (2020). Geneious R10 (BioMatters, Ltd.) was used for all sequence inspection, editing, assembly, and alignment. Sequence ends were automatically trimmed (2.5% error limit) followed by manual inspection. Consensus sequences for each sample were aligned with the MAFFT plug-in. Clone identity was determined using NCBI BLAST (default megablast).

## Results

### Gut Microbiome Diversity and Structure

**FIGURE 2:**
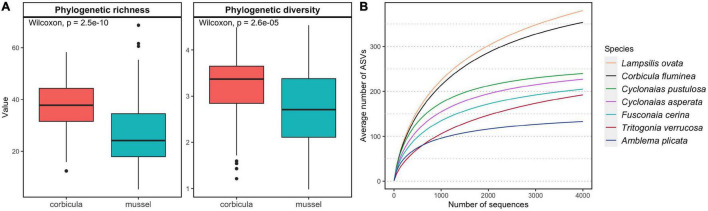
Alpha diversity of the gut microbiomes of six native freshwater mussel species and the invasive clam *Corbicula fluminea*, collected from six rivers in the Tennessee and Mobile River Basins, United States. **(A)** Distribution of phylogenetic richness (Faith’s PD) and phylogenetic diversity (Allen index of diversity) in *C. fluminea* and mussels. **(B)** Average accumulation curves representing the number of amplified sequence variants (ASVs) in random subsamples of 1 to 4000 sequences per sample. Other descriptors of alpha diversity and coverage are available in S2-Supplementary Table 1.

Phylogenetic richness based on presence-absence data and diversity weighted by relative abundances of ASVs were higher in *C. fluminea* microbiome than that of native mussels (Figure 2 and S2-Supplementary Table 1, Wilcoxon tests, *P* < 0.001). The *C. fluminea* microbiome was richer phylogenetically and taxonomically than every co-occurring mussel species sampled, except for that of *L. ovata*, which reached similar levels (Figure 2 and S2-Supplementary Table 1). Phylogenetic and taxonomic and diversity accounting for relative abundance were also significantly higher in *C. fluminea* when compared to two and four of the native mussel species, respectively (S2-Supplementary Table 1). Conversely, estimated coverage was significantly higher for every mussel species compared to *C. fluminea*, excepted for L. ovata (S2-Supplementary Table 1). While *C. fluminea* shared 21.4-26.9% of its ASVs with co-occurring mussel species, each mussel species shared 31.5-53.1% of their ASVs with co-occurring *C. fluminea*.

**FIGURE 3:**
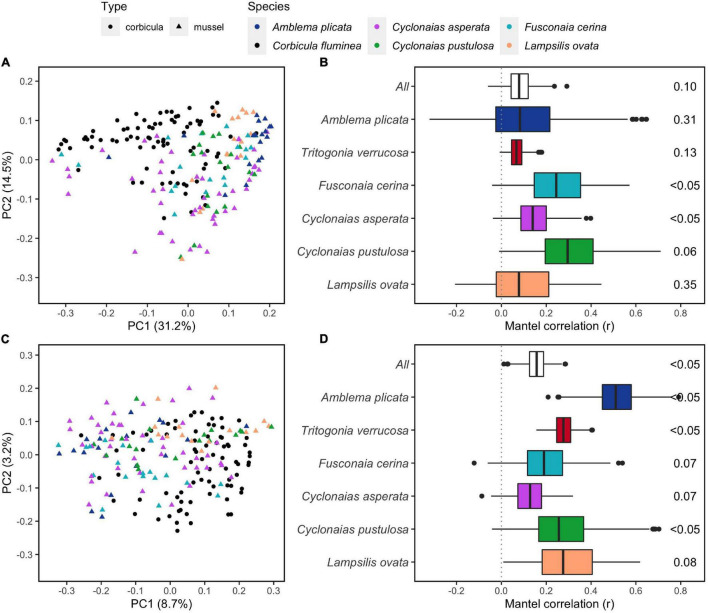
Dissimilarities between gut microbiomes of six native freshwater mussel species and the invasive clam *Corbicula fluminea*, collected from rivers in the Tennessee and Mobile River Basins. A and C: Principal Coordinate Analyses (PCoAs) representing the weighted **(A)** and unweighted **(C)** versions of Unifrac phylogenetic dissimilarity between individual microbiomes of five of the mussel species, and *C. fluminea* (PCoAs with all six species including *T. verrucosa* are available in S2-Supplementary Figure 1). B and D: correlations between dissimilarities of W-Unifrac **(B)** and U-Unifrac **(D)** of *C. fluminea* and mussel microbiomes, assessed by 500 Mantel tests on random subsamples of the same number of specimens at each site (either with all mussel species – “All,” or per species). The median of the 500 *p*-values, indicating an overall significant co-variation between *C. fluminea* and mussel microbiomes, is displayed on the right.

The overall structure of the *C. fluminea* gut microbiome was distinct from that of native mussels as a group (PERMANOVA, *P* = 0.001, R^2^ = 0.09 and 0.03 for W- and U-Unifrac, respectively, Figures 3A,C); more distinct from seston bacterial communities (*P* = 0.001, R^2^ = 0.17 for both indices); and even more distinct from sediment bacterial communities (*P* = 0.001, R^2^ = 0.47 and 0.50). The structure of the *C. fluminea* microbiome and that of each mussel species were systematically distinct, especially based on W-Unifrac (R^2^ = 0.11-0.33 depending on the mussel species considered; S2-Supplementary Table 2). The mussels *T. verrucosa* and *A. plicata* hosted the most distinctive microbiomes compared to that of *C. fluminea*, and *T. verrucosa* sampled from the Buttahatchee and Sipsey Rivers exhibited the most unique microbiome structure compared to all other species (Figure 3 and S2-Supplementary Table 2, S2-Supplementary Figure 1). Variability in microbiome structure was equivalent for *C. fluminea* and each mussel species, other than greater variability in *T. verrucosa* and *C. asperata* (permutation tests on betadisper, *P* < 0.05).

The mean dissimilarity between *C. fluminea* and native mussels (U-Unifrac = 0.73 ± 0.06; W-Unifrac = 0.36 ± 0.10) was similar to that between native mussel species (U-Unifrac = 0.73 ± 0.06; W-Unifrac = 0.39 ± 0.12). When the analyses were performed excluding *T. verrucosa*, to eliminate the greater influence of this mussel species, *C. fluminea* vs. mussel dissimilarity (U-Unifrac = 0.73 ± 0.04; W-Unifrac = 0.33 ± 0.08) remained similar to that within native mussel species (U-Unifrac = 0.73 ± 0.05; W-Unifrac = 0.34 ± 0.08).

Additionally, the gut microbiome of *C. fluminea* tended to co-vary with that of native mussels, especially based on the presence-absence of ASVs (500 Mantel tests on U-Unifrac, median P < 0.1, Figure 3). However, this correlation was not significant in *T. verrucosa*, *A. plicata*, and *L. ovata* when considering relative abundance weighted dissimilarities (median *P* > 0.1, Figure 3).

### Geographic Variation in the Gut Microbiome

**FIGURE 4:**
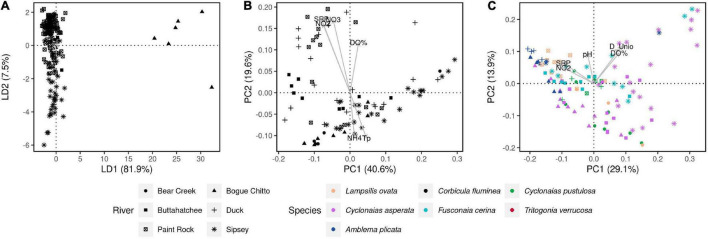
Genetic and microbial dissimilarities in the invasive clam *C. fluminea* and six native freshwater mussel species, collected from six rivers in the Tennessee and Mobile River Basins. **(A)** DAPC showing the genetic dissimilarities between all 173 *C. fluminea* specimens that were included in the population genomics analysis (RADSeq). **(B)** PCoA displaying W-Unifrac dissimilarities between the gut microbiomes of the 80 individuals analyzed for their microbiome. **(C)** PCoA displaying W-Unifrac dissimilarities between the gut microbiomes of the native freshwater mussels collected. On plots **(B,C)**, Physicochemical and biological variables were correlated to the coordinates of each individual microbiome and are represented by arrows. Only parameters showing a significant correlation (*P* < 0.05) are shown. D_Unio: unionid density; D_Corb: *C. fluminea* density; S: unionid richness; NO2: water nitrite; NO3: water nitrate; SRP: soluble reactive phosphorus in water; Tp: water temperature.

Structures of *C. fluminea* and native mussel microbiomes varied across rivers and sampling sites, and these effects were higher for W-Unifrac than U-Unifrac (PERMANOVAs, *P* < 0.01 and R^2^ = 0.14-0.38 for every mussel species, and *P* < 0.01 and *C. fluminea*, R^2^ = 0.15-0.58, respectively; S2-Supplementary Table 3). Microbiome dissimilarities of *C. fluminea* showed a positive correlation with river distance, i.e., more distant individuals along the same river tended to have more distinct microbiomes (Spearman test, *P* < 0.001, S2-Supplementary Figure 2). Among environmental variables, soluble nitrate (*P* = 0.001, R^2^ = 0.40), nitrite (*P* = 0.001, R^2^ = 0.32), SRP (*P* = 0.02, R^2^ = 0.14), water temperature (*P* = 0.02, R^2^ = 0.14) and dissolved oxygen (*P* = 0.03, R^2^ = 0.12), were significantly correlated to variations in the structure of *C. fluminea* microbiome (Figure 4).

The microbiome dissimilarities between native mussels (other than *T. verrucosa*) were correlated to unionid density (*P* = 0.002, R^2^ = 0.23), dissolved oxygen (*P* = 0.002, R^2^ = 0.17), nitrite (*P* = 0.001, R^2^ = 0.17), SRP (*P* = 0.005, R^2^ = 0.13), pH (*P* = 0.003, R^2^ = 0.12), and water temperature (*P* = 0.02, R^2^ = 0.08) (envfit, Figure 4). The microbiome dissimilarities between *T. verrucosa* only correlated to SRP (*P* = 0.02, R^2^ = 0.22). There was no significant correlation between *C. fluminea* density and the microbiome dissimilarities between native mussels (envfit, *P* = 0.38).

### Population Genomic Variation in *Corbicula fluminea* and Its Relationship to the Gut Microbiome

DNA barcoding with COI confirmed that samples were identical to the *C. fluminea* lineage “A” (S3-Supplementary Figure 1), the most common in North America. Clonal lineage thus does not appear to have a major contribution to microbiome dissimilarities across *C. fluminea* individuals in these rivers. 3,580,850 bp were sequenced by RAD-tag sequencing to a mean coverage of 44.15x per site sample, with a mean of 2.4% missing data per sample. Nucleotide diversity was similar among populations (π = 0.0049 - 0.0053 for all rivers) and thus was also not considered a major predictor of microbiome variation (S3-Supplementary Table 1). Consistent with extensive clonal reproduction (Haponski and Foighil, 2019), FIS was strongly negative in all populations (FIS values −0.92 to −0.85, S3-Supplementary Table 1), and there were many SNPs (7,213 of 34,646 SNPs) that were heterozygotes across all sequenced individuals. After removing these sites and thinning to one SNP per kb, 5,225 SNPs remained for analysis of population structure. Pairwise FST showed weak but significant differentiation of *C. fluminea* across rivers (S3-Supplementary Table 2). DAPC analysis also indicated within clonal lineage variation in diversity that was primarily structured by river, although not by basin; samples from the Bogue Chitto River largely separated on Axis 1 and Axis 2 transitioned from Sipsey River samples (negatively loading) to Paint Rock and Bear Creek River samples, to Duck and Buttahatchee River samples (positively loading) (Figure 4). Within rivers, genetic dissimilarities of *C. fluminea* exhibited a positive correlation with geographical distance, i.e., more distant individuals tended to have more distinct genomes (Spearman correlation testS, *P* < 0.001, S2-Supplementary Figure 2). Despite this structuring of *C. fluminea* genetic diversity and geographic variation in gut microbiomes, no significant correlations between genetic distances and microbiome dissimilarities were detected, either globally (Mantel tests on W- and U-Unifrac and individual genetic dissimilarities, *P* > 0.05) or within rivers (Spearman’s correlation test on dissimilarities between individuals collected along the same river, *P* = 0.02, *r* = −0.1, Figure 4 and S2-Supplementary Figure 2). Similarly, no correlation was detected based on FST between rivers (Mantel tests on averaged W- and U-Unifrac per river and FST, *P* > 0.05).

### Composition

The *C. fluminea* gut microbiome consisted of 69 different bacterial phyla and 126 bacterial classes, with the classes accounting for the greatest proportion of sequences being similar to those in the microbiome of the six native mussel species. These included Clostridia (34.7 ± 21.2% and 14.1 ± 20.3% of the sequences for *C. fluminea* and native mussels, respectively), Planctomycetacia (18.1 ± 9.3% and 23.5 ± 15.3%), Alphaproteobacteria (10.6 ± 5.1% and 12.7 ± 8.9%), Gammaproteobacteria (9.7 ± 10.6% and 8.2 ± 7.4%), and Bacilli (4.3 ± 4.9% and 3.5 ± 5.7%). Native mussels also presented high percentages of Mollicutes, which were of a lower abundance in *C. fluminea* (12.8 ± 14.9% in mussels vs. 4.5 ± 5.5%). Compared to all species of native mussels and across all sites, the *C. fluminea* microbiome contained higher proportions of Actinobacteriota (LefSE log10 LDA score = 3.0), Verrucomicrobiota (2.9), and Planctomycetes (3.5), especially the Gemmataceae (3.2) and Pirellulaceae (3.0). Eleven of the most abundant ASVs were found to be significantly enriched in *C. fluminea* compared to native mussels, the most numerous ones classified as members of genera *Romboutsia* (Clostridiales), *Stenotrophomonas* (Xanthomonadales), *Epulopiscium* (Lachnospirales), and *Paraclostridium* (Peptostreptococcales) (S2-Supplementary Figure 3).

### Potential Reciprocal Influences of *Corbicula fluminea* and Mussel Microbiomes

**FIGURE 5:**
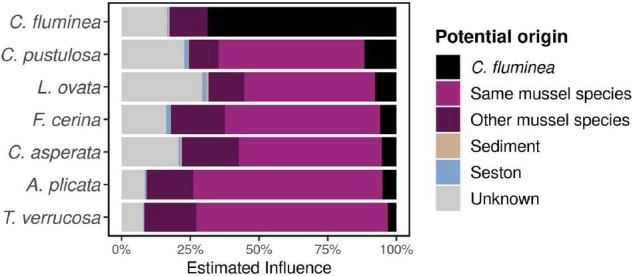
Influence of co-occurring host-associated, seston, and sediment microbes over individual microbiomes, estimated using FEAST software and averaged per species. Globally, individual bivalves presenting a higher reciprocal influence (native mussels over *C. fluminea*, and reversely) presented a higher phylogenetic richness (Pearson’s correlation test on log-transformed data, *P* < 0.001, *r* = 0.6).

The estimated influence of the *C. fluminea* microbiome on that of the co-occurring mussels was on average of 5.8 ± 8.0%, while the influence of the native mussel microbiome on the *C. fluminea* microbiome was 14.0 ± 12.2% (KW on FEAST results, *P* < 0.001) (Figure 5). The estimated influence of the *C. fluminea* microbiome over that of native mussels was primarily dependent on the recipient mussel species (KW, *P* < 0.001), and independent from the collection site (KW, *P* = 0.08). Mussel species that were under the greatest influence from the *C. fluminea* microbiome were *Cyclonaias pustulosa* (11.6 ± 13.5%) and *Lampsilis ovata* (7.4 ± 7.7%), while the species that was the least influenced was *Tritogonia verrucosa* (2.4 ± 3.8%).

In contrast, the influence of the native mussel microbiome on that of *C. fluminea* was significantly distinct across sites, varying from 4.67% on site FAY (Sipsey river) to 15.3% on BTN (Paint Rock river, KW, *P* = 0.01), but not across source mussel species (KW, *P* = 0.051, varying from 4.39% from *T. verrucosa* to 9.47% from *C. pustulosa* although no mussel species were sampled on every site, making such comparison difficult, Figure 1 and S1-Supplementary Table 1). However, neither water physicochemical characteristics nor biotic variables (*C. fluminea* and unionid densities) explained such variation of mussel contribution across sites variation (Spearman’s correlation tests, *P* > 0.05).

### Inferred Functions and Degradation Potential

**FIGURE 6:**
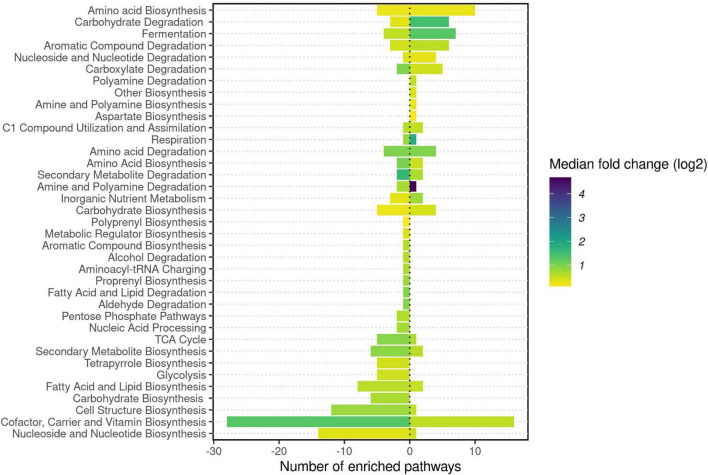
DESeq2 analysis of inferred microbial functions from 16S rRNA gene data of six freshwater mussel species and the invasive clam Corbicula fluminea collected from six rivers in the Tennessee and Mobile River Basins. DESeq2 was used to determine which inferred pathways were significantly enriched in *C. fluminea* compared to native mussels, before the class of each pathway was recorded from the MetaCyc database and the number of pathways significantly enriched in each class was reported. Pathways enriched in *C. fluminea* are presented by positive values, while the negative values represent those enriched in mussels. For each host type and each class of pathway, the median of the log2 Fold change was used to color the bars.

The 436 functional pathways were inferred from the *C. fluminea* gut microbiome sequence data compared to 443 for native mussels. The functional potential of *C. fluminea* was distinct overall from that of native mussels (PERMANOVA based on Bray-Curtis, *P* = 0.001, R^2^ = 0.07), especially from that of *T. verrucosa*, *A. plicata*, and *C. asperata* (PERMANOVA’s pairwise *post hoc* tests, *P* < 0.05). Compared to native mussels, the functional potential of the *C. fluminea* gut microbiome was enriched in pathways related to the degradation of various compounds (especially carbohydrates and carboxylates, aromatic compounds). In contrast, the microbiome of mussels included higher potential for biosynthesis (especially cofactors and vitamins, nucleotides, and nucleosides) (Figure 6). Among the seven pathways that contributed the most to the differences between the *C. fluminea* and mussel microbiomes, six were classified as a degradation or assimilation function, and only one to biosynthesis (methylquinone). All seven revealed a potential for higher expression in *C. fluminea* than in native mussels (S2-Supplementary Figure 4). Accordingly, the degradation potential of each microbiome, taking into account all degradation pathways, was significantly higher for *C. fluminea* than for any native mussel species except *L. ovata*, where it reached similar levels (S2-Supplementary Figure 5).

## Discussion

Here we document for the first time the gut microbiome of the invasive *C. fluminea*. We demonstrate that while this species’ microbiome is diverse, it is not highly distinctive from that of the co-occurring native mussels. The high correlation of the structure of the *C. fluminea* microbiome with local environmental parameters, together with the influence of co-occurring mussel species and the lack of influence of host genetic ancestry, suggests that the microbiome of this invasive species may be largely dependent on extrinsic factors such as environmental conditions and local bacterial occurrence.

### Differences in the Microbiome Between *Corbicula fluminea* and Native Mussels

The microbiome structure of *C. fluminea* was only partially distinct from that of six co-occurring native mussel species, with similar dominant classes of Bacteria but differences occurring at a finer taxonomic level. Overall similarities between the microbiome of native mussels and *C. fluminea* could be explained by the trophic similarity between these organisms, primarily filter-feeding on suspended particles and deposit-feeding within the sediment (Sousa et al., 2008; Schartum et al., 2017). However, given the species-specificity of microbiomes within freshwater mussels (i.e., distinct species occupying the same habitat host distinct microbiomes, (Weingarten et al., 2019; McCauley et al., 2021), we expected a more distinct *C. fluminea* microbiome given its contrasting phylogeny and life history traits to those of freshwater mussels. We also found no evidence that intra-specific genetic ancestry or diversity was related to microbiome variation within *C. fluminea*. All individuals sampled belong to the clonal lineage A (Haponski and Foighil, 2019) and although SNP data revealed significant within-lineage genetic differentiation across rivers, there was no effect of genetic differentiation among individuals or populations on their microbiomes. Instead, the structure of *C. fluminea* microbiome was tightly correlated to local environmental variables, with nitrates, nitrites, and SRP being the primary drivers.

Studies on other invertebrates have demonstrated a consistent effect of host genotype on the microbiome when hosts are reared under controlled or germ-free conditions (Wegner et al., 2013; Callens et al., 2020), so that it is likely that the lack of correlation between the genotype of *C. fluminea* and its gut microbiome in this study is related to variable field conditions. Further studies under controlled conditions would be needed to confirm the absence of a host genotype-microbiome link in this aquatic filter-feeder.

### Diversity of the Microbiome of *Corbicula fluminea* and Inferred Functions

We observed a greater microbial richness in the gut microbiome of *C. fluminea* compared to co-occurring native mussels, except for *L. ovata*, which had similar levels of microbiome diversity. Such higher diversity may be related to differences in feeding behavior, as a higher clearance rate or a lower selectivity in ingested particles could induce gut colonization by a greater diversity of bacteria. *C. fluminea* has a higher filter-feeding rate than the few native mussels that have been studied so far (Hills et al., 2020; Pouil et al., 2021), and while systematic comparisons are lacking, *C. fluminea* also shows a low prey selectivity (seemingly limited to an avoidance of toxic bacterioplankton (Bolam et al., 2019) and assimilates a wider range of substrates (Atkinson et al., 2010). Unionid mussels exhibit a higher degree of filtration selection and a narrower range of substrate assimilation (Beck and Neves, 2003; Atkinson et al., 2010; Lopes-Lima et al., 2014b). The greater bacterial diversity hosted by *C. fluminea* may allow them to consume a broader range of nutritional sources. This could provide a competitive advantage for *C. fluminea* and, accordingly, functional inferences predicted a greater diversity of degradation potential in the gut microbiome of *C. fluminea* compared to native mussels, and most metabolic pathways that were enriched in *C. fluminea* were associated with degradative functions. This hypothesis is based on functional inferences and should be studied further with appropriate metagenomic analysis of the *C. fluminea* gut microbiome to confirm such higher prevalence of degradation functions, combined with experiments to assess the relationship between the *C. fluminea* microbiome and the nature of successfully digested particles.

### Reciprocal Influences Between Invasive and Native Species

Overall, native mussels shared a substantial fraction of their microbiomes with co-occurring *C. fluminea* (31.5-53.1% of ASVs depending on mussel species), and *C. fluminea* and native mussel microbiomes tended to co-vary across sites. The FEAST estimate of mussel influence over co-occurring *C. fluminea* microbiome was more than twice that of the reverse interaction. This may indicate that *C. fluminea* may host gut populations that are obtained locally, including from native mussels that may be indirectly transferred through the water column or the sediment *via* feces and pseudofeces. While such potential contamination should be further explored in controlled conditions, it may confirm evidence from plants that suggests that invasive species can suffer from the loss of their natural bacterial symbionts when they are introduced to a new environment, and must develop novel interactions with local bacteria, potentially bacteria associated with co-occurring native species (Parker et al., 2006; Shelby et al., 2016).

Accordingly, while comparisons of the microbiome of invasive animal species across native vs. introduced range are lacking, a study on invasive tunicates documented a more variable and more diverse microbiome in individuals in the introduced range than those in their native range, which may also suggest this hypothesis could be confirmed in invasive animals (Utermann et al., 2020). Moreover, in our study, while the estimated influence of mussel microbiome over that of *C. fluminea* was variable across sites, it was independent of local unionid density. Although appropriate density experiments must confirm this, this may indicate that even low densities of native species may provide microbial partners to *C. fluminea* microbiome.

## Conclusion

We show that the gut microbiome of the invasive clam *C. fluminea* presents several characteristics (e.g., higher diversity, higher functional potential, and potentially a lower selectivity and a higher rate of horizontal transmission from native counterparts) that may be beneficial for such a globally invasive organism to acclimate to a non-native area. This work should be further developed, focusing on understanding which bacterial partners may be more beneficial to *C. fluminea* to assess whether these partners are also enriched in populations of *C. fluminea* in their native range, or are indicative of invasive areas. Studies evaluating geographic regions with additional clonal lineages would also be valuable for fully understanding how genetic diversity contributes to microbiome variation in this invasive species. Focused analyses could pave the way for a more nuanced understanding of the transport of symbionts within invasive animal species, which is still a poorly investigated topic, and has the potential to develop pre- or probiotics for endangered native species.

## Data Availability Statement

The datasets presented in this study can be found in online repositories. The names of the repository/repositories and accession number(s) can be found below: https://www.ncbi.nlm.nih.gov/genbank/, OK047371-OK047417; https://www.ncbi.nlm.nih.gov/sra, PRJNA757758, PRJNA757734, PRJNA740316, and PRJNA761344.

## Author Contributions

MC and CJ conceptualized the study. CA, GH, IS, JB, MM, and MC participated in sample collection and fieldwork. MC and SV processed the microbiome samples and prepared the libraries.MC analyzed the microbiome data. JB and JL performed RADseq and DNA barcoding and analyzed the data. MC wrote the manuscript, with contributions from JB and JL for the genomic analyses. All authors read and approved the final manuscript.

## Conflict of Interest

The authors declare that the research was conducted in the absence of any commercial or financial relationships that could be construed as a potential conflict of interest.

## Publisher’s Note

All claims expressed in this article are solely those of the authors and do not necessarily represent those of their affiliated organizations, or those of the publisher, the editors and the reviewers. Any product that may be evaluated in this article, or claim that may be made by its manufacturer, is not guaranteed or endorsed by the publisher.

## Footnotes

1
https://www.invasivespeciesinfo.gov/

2
https://earthmicrobiome.org/protocols-and-standards/16s/

3
https://benjjneb.github.io/dada2/tutorial.html

4
https://github.com/pmartinezarbizu/pairwiseAdonis

5
http://broadinstitute.github.io/picard
