# Supplementary material for: Environment and Co-occurring Native Mussel Species, but Not Host Genetics, Impact the Microbiome of a Freshwater Invasive Species (Corbicula fluminea)
Source: Front Microbiol. 2022 Apr 4;13:800061. doi: 10.3389/fmicb.2022.800061 (PMC9014210; doi:10.3389/fmicb.2022.800061)
Supplement: Supplementary file 1 [file Data_Sheet_1.DOCX]

Supplementary ***Information S1: Collection sites***

**S1-Table 1**: Number of *C. fluminea* and native freshwater mussels collected from 16 sites on six rivers in the Mobile and Tennessee River Basins (n mussels and *C. fluminea* collected). For each mussel species, 3-10 individuals were collected on each site, with varying number of species per site. **C. fluminea* collected on sites;***C. fluminea* included in microbiome analysis; ****C. fluminea* included in RADSeq analysis.

| **River** | **Site** | **n Mussels** | **n Mussel species** | **n *C. fluminea* collected*** | **n *C. fluminea* (microbiome)**** | **n *C. fluminea* (RADSeq)***** | **Sampling date** |
| --- | --- | --- | --- | --- | --- | --- | --- |
| Bogue Chitto | BOG | 15 | 2 | 3 | 3 | 2 | 2019-07-18 |
|  | BIS | 5 | 1 | 4 | 4 | 4 | 2019-07-19 |
| Buttahatchee | BUT | 19 | 3 | 5 | 4 | 5 | 2019-08-20 |
|  | BBT | 18 | 3 | 5 | 5 | 5 | 2019-08-16 |
| Sipsey | FAY | 3 | 1 | 5 | 5 | 5 | 2019-08-13 |
|  | MUS | 18 | 3 | 4 | 4 | 4 | 2019-07-12 |
|  | WEN | 14 | 3 | 8 | 8 | 7 | 2019-08-22 |
|  | DRN | 15 | 3 | 25 | 7 | 25 | 2019-09-19 |
| Bear Creek | BON | 12 | 2 | 5 | 5 | 1 | 2019-07-30 |
| Duck | COL | 7 | 2 | 26 | 5 | 26 | 2019-09-05 |
|  | HOP | 0 | 0 | 28 | 5 | 28 | 2019-09-05 |
|  | LIL | 0 | 0 | 27 | 5 | 27 | 2019-09-05 |
|  | VEN | 0 | 0 | 20 | 5 | 20 | 2019-09-05 |
| Paint Rock | BTN | 4 | 1 | 5 | 5 | 5 | 2019-07-24 |
|  | FIS | 10 | 2 | 5 | 5 | 4 | 2019-07-25 |
|  | JON | 4 | 1 | 5 | 5 | 5 | 2019-07-23 |
|  | TOTAL | 144 | 6 | 180 | 80 | 173 |  |

**S1-Table 2**: Physico-chemical characteristics of the water and granulometry of surface sediment on sampling sites. Dissolved Organic Carbon (DOC), dissolved Ammonium (NH4+), soluble reactive phosphorous (SRP) and temperature have been recorded at the beginning of each sampling day. Water conductivity, pH, dissolved oxygen (DO%), nitrates (NO3-) and nitrites (NO2-) have been assessed in June 2019 ahead of sampling. NH4+, NO3- and NO2- were measured like in Nickerson et al (2019). DOC and SRP were assessed like in Hooper et al (2021). Water DO%, pH, temperature and conductivity were measured using a multiparameter probe (YSI Inc., Yellow Springs, OH). Sediment granulometry was assessed by measuring randomly 93-231 particles of surface sediment (depending on the number of quadrats per site), and described as D16, D50 and D84, as the class of size below which 16%, 50% and 84% of the measured material is contained, respectively.

| **River** | **Site** | **DOC  (µmol/L)** | **NH4+  (µmol /L)** | **SRP  (µmol /L)** | **Water temperature (°C)** | **Sediment granulometry** | | | **DO%** | **NO2-  (µg /L)** | **NO3-  (µg /L)** |
| --- | --- | --- | --- | --- | --- | --- | --- | --- | --- | --- | --- |
|  |  |  |  |  |  | D16 | D50 | D84 |  |  |  |
| Bogue Chitto | BOG | 519.8 | 1.28 | 0,45 | 29.3 | 2 | 11 | 22.6 | 77.5 | 3.00 | 29.25 |
|  | BIS | 482.5 | 0.75 | 0,44 | 28.8 | 4 | 11 | 22.6 | 92.2 | 3.50 | 17.08 |
| Buttahatchee | BUT | 391.3 | 0.39 | 0,25 | 27.3 | 8 | 16 | 22.6 | 85.8 | 3.62 | 349.30 |
|  | BBT | 461.2 | 0.42 | 0,19 | 28.6 | 5.6 | 11 | 22.6 | 85 | 2.62 | 415.57 |
| Sipsey | FAY | 230.8 | 1.60 | 0,25 | 26.1 | 2 | 8 | 11 | 82 | 3.42 | 297.84 |
|  | MUS | 248.9 | 1.59 | 0,07 | 31.1 | 4 | 11 | 16 | 87.5 | 3.13 | 138.94 |
|  | WEN | 775.3 | 0.91 | 0,23 | 29.4 | 2 | 8 | 16 | 92.8 | 1.87 | 108.59 |
|  | DRN | 206.2 | 0.34 | 0,33 | 28.7 | 2 | 8 | 16 | 93.3 | 2.84 | 266.86 |
| Bear creek | BON | 169.7 | 0.50 | 0,29 | 27.2 | 2.8 | 16 | 22.6 | 87.8 | 3.27 | 283.82 |
| Duck | COL | 200 | 0.81 | 2,57 | 24.7 | 4 | 11 | 22.6 | 92.5 | 9.98 | 416.31 |
|  | HOP | - | - | - | - | 5.6 | 16 | 32 | - | 4.31 | 615.85 |
|  | LIL | - | - | - | - | 5.6 | 16 | 22.6 | - | 9.27 | 592.79 |
|  | VEN | - | - | - | - | 5.6 | 11 | 22.6 | - | 6.53 | 589.31 |
| Paint Rock | BTN | 157.5 | 1.28 | 0,28 | 25.0 | 2 | 11 | 22.6 | 80 | 6.61 | 737.35 |
|  | FIS | 155.4 | 0.77 | 0,33 | 25.2 | 2 | 11 | 22.6 | - | 4.36 | 640.03 |
|  | JON | 167.0 | 0.44 | 0,28 | 28.9 | 2 | 16 | 32 | - | 3.96 | 311.33 |

**Bibliography:**

Nickerson, Z. L., Mortazavi, B., & Atkinson, C. L. (2019). Using functional traits to assess the influence of burrowing bivalves on nitrogen-removal in streams. *Biogeochemistry*, *146*(2), 125-143.Hopper, G. W., Chen, S., Sanchez Gonzalez, I., Bucholz, J. R., Lu, Y., & Atkinson, C. L. (2021). Aggregated filter‐feeders govern the flux and stoichiometry of locally available energy and nutrients in rivers. *Functional Ecology*, *35*(5), 1183-1195.
