# Supplementary material for: Environment and Co-occurring Native Mussel Species, but Not Host Genetics, Impact the Microbiome of a Freshwater Invasive Species (Corbicula fluminea)
Source: Front Microbiol. 2022 Apr 4;13:800061. doi: 10.3389/fmicb.2022.800061 (PMC9014210; doi:10.3389/fmicb.2022.800061)
Supplement: Supplementary file 2 [file Data_Sheet_2.DOCX]

Supplementary ***Information S2: Supplementary Microbiome Results***

# **S2-Supplementary Tables**

**S2-Table 1**: Descriptors of taxonomic and phylogenetic alpha-diversity and coverage of the gut microbiomes of six native freshwater mussel species and the invasive clam *Corbicula fluminea* collected from six rivers in the Tennessee and Mobile River Basins, southeastern US. Taxonomic richness: number of observed amplified sequence variants (ASVs) in each sample; taxonomic diversity: Shannon index expressed as equivalent number of species; phylogenetic richness: Faith’s PD; phylogenetic diversity: index of Allen; Coverage: Chao’s coverage. All values were indicated as Mean ± Standard Deviation for each species. Alpha-diversity metrics were compared to those of *C. fluminea* occurring at the same sites, and results of the pairwise comparison are displayed for each species and measurement. *P<0.05; **P<0.01; ***P<0.001.

| Species | Taxonomic richness | Taxonomic diversity | Phylogenetic richness | Phylogenetic diversity | Coverage |
| --- | --- | --- | --- | --- | --- |
| *C. fluminea* | 353±140 | 96.2±55.2 | 37.3±10.3 | 3.2±0.7 | 0.98±0.0 |
| *L. ovata* | 380±242 | 96.2±55.2 | 37.8±14.0 | 3.5±0.7 | 0.97±0.0 |
| *C. pustulosa* | 239±102(*) | 76.9±51.9 | 29.5±10.3(*) | 3.2±0.8 | 0.99±0.0(**) |
| *C. asperata* | 227±126(***) | 57.5±49.1(***) | 27.8±10.4(***) | 2.9±0.8 | 0.99±0.0(***) |
| *F. cerina* | 205±121(***) | 43.6±28.0(**) | 26.1±9.7(**) | 2.7±0.7 | 0.99±0.0(**) |
| *T. verrucosa* | 192±202(***) | 25.4±37.7(***) | 22.2±15.6(***) | 2.1±0.9(***) | 0.98±0.0(**) |
| *A. plicata* | 133±47(***) | 29.6±15.8(***) | 19.4±5.0(***) | 2.4±0.6(***) | 0.99±0.0(***) |

**S2-Table 2:** Distinction of microbiome community structure between *C. fluminea* and each of the 6 mussel species included in this study, assessed using separated PERMANOVAs based on U- and W-Unifrac. P: P-value of the test after 999 permutations; R^2^: R-squared value, indicating how much of the variability in microbiome structure is explained by the distinction between *C. fluminea* microbiome and the one of the mussel species included in the test.

|  | **U-Unifrac** | | **W-Unifrac** | |
| --- | --- | --- | --- | --- |
| **Mussel species** | **P** | **R^2^** | **P** | **R^2^** |
| *Amblema plicata* | 0.001 | 0.05 | 0.001 | 0.21 |
| *Tritogonia verrucosa* | 0.001 | 0.07 | 0.001 | 0.33 |
| *Fusconaia cerina* | 0.001 | 0.04 | 0.001 | 0.09 |
| *Cyclonaias asperata* | 0.001 | 0.04 | 0.001 | 0.11 |
| *Cyclonaias pustulosa* | 0.001 | 0.02 | 0.001 | 0.12 |
| *Lampsilis ovata* | 0.001 | 0.02 | 0.001 | 0.11 |

**S2-Table 3**: Correlation of microbiome dissimilarities and sampling river and sites, assessed separately for *C. fluminea* and each mussel species studied using separated PERMANOVAs based on U- and W-Unifrac. P: P-value of the test after 999 permutations; R^2^: R-squared value, indicating how much of the variability in microbiome structure is explained by the distinction between sites or rivers.

|  |  | **U-Unifrac** | | **W-Unifrac** | |
| --- | --- | --- | --- | --- | --- |
| **Species** | **Factor** | **P** | **R^2^** | **P** | **R^2^** |
| *A. plicata* | River | 0.001 | 0.31 | 0.001 | 0.37 |
|  | Site | 0.001 | 0.31 | 0.001 | 0.37 |
| *C. fluminea* | River | 0.001 | 0.15 | 0.001 | 0.25 |
|  | Site | 0.001 | 0.32 | 0.001 | 0.58 |
| *C. asperata* | River | 0.001 | 0.11 | 0.001 | 0.25 |
|  | Site | 0.001 | 0.24 | 0.001 | 0.35 |
| *C. pustulosa* | River | 0.001 | 0.21 | 0.001 | 0.38 |
|  | Site | 0.001 | 0.21 | 0.001 | 0.38 |
| *F. cerina* | River | 0.006 | 0.08 | 0.005 | 0.19 |
|  | Site | 0.001 | 0.22 | 0.011 | 0.31 |
| *L. ovata* | River | 0.004 | 0.14 | 0.007 | 0.27 |
|  | Site | 0.002 | 0.23 | 0.002 | 0.33 |
| *T. verrucosa* | River | 0.001 | 0.16 | 0.005 | 0.20 |
|  | Site | 0.001 | 0.25 | 0.009 | 0.30 |

# S2-**
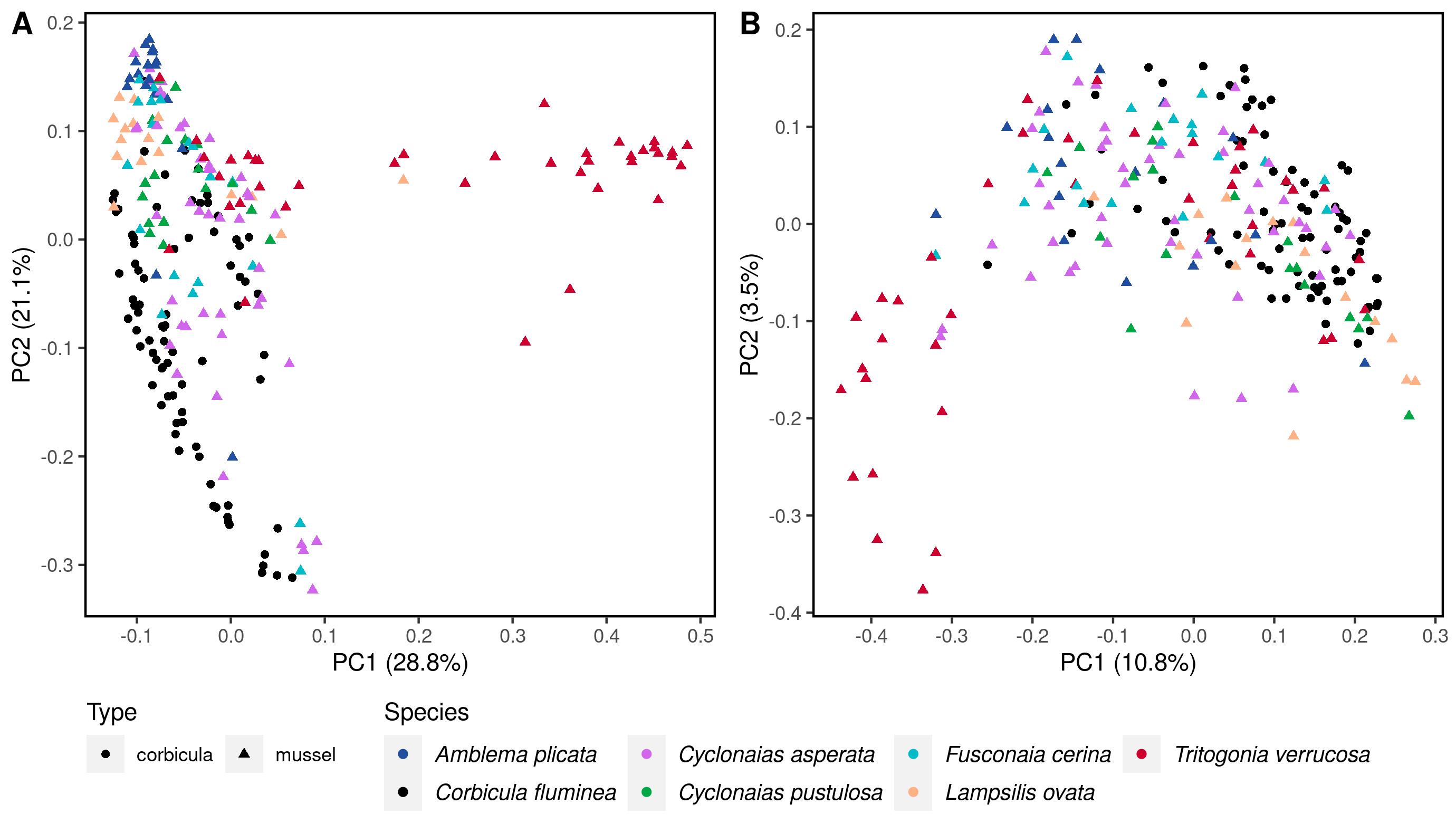
Supplementary Figures**

**S2-Figure 1:** Principal Coordinate Analyses (PCoAs) representing the dissimilarities between gut microbiomes of six native freshwater mussel species and the invasive clam *Corbicula fluminea*, collected from six rivers inthe Tennessee and Mobile River Basins. A: Weighted Unifrac (W-Unifrac) and B: Unweighted Unifrac (U-Unifrac). The group of *T. verrucosa* samples that were highly divergent from other samples were collected from the Sispey and Buttahatchee Rivers, while *T. verrucosa* that were more similar to the rest of samples came from the Paint Rock River.


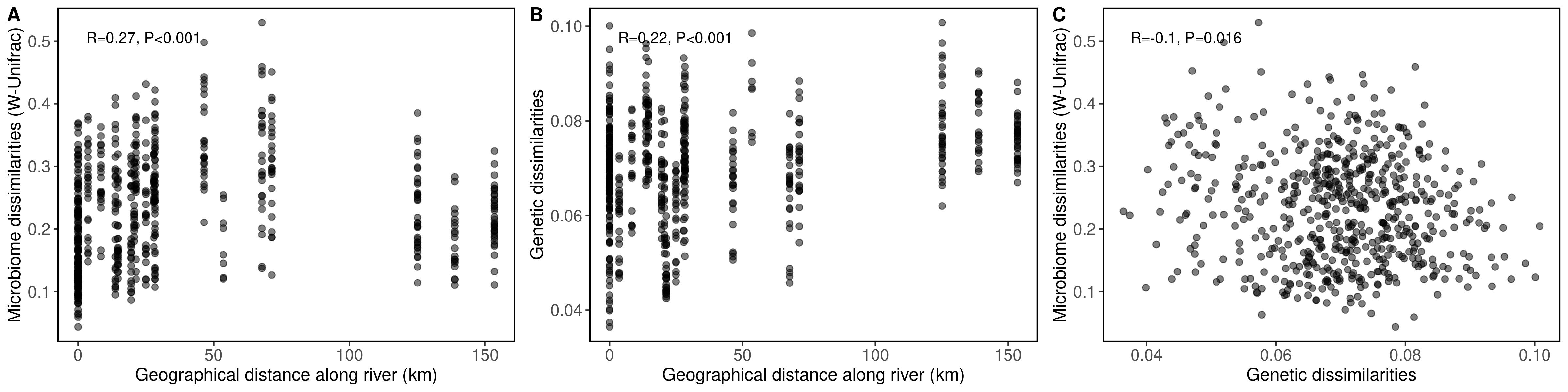
**S2-Figure 2**: Correlation between gut microbiome variation, geographical distances along river, and genomic data from C. fluminea. A: Correlation between microbiome dissimilarities assessed by W-Unifrac, and geographical distances, on specimens collected along the same river. B: Correlation between genetic dissimilarities and geographical distances. C: Correlation between genetic dissimilarities and microbiome dissimilarities. Significant correlations were assessed using Spearman correlation tests, which result is indicated on each plot.


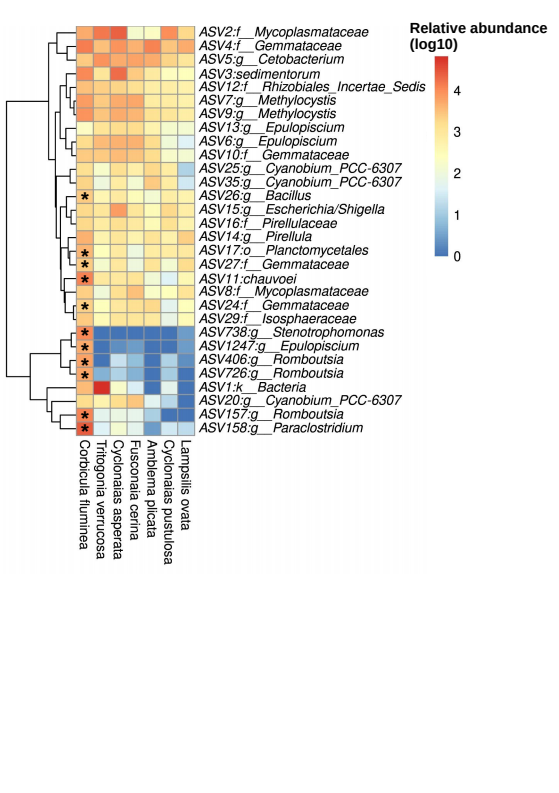
**S2-Figure 3**: Heatmap showing the log-transformed relative abundance of the 30 proportionally most abundant amplified sequence variants (ASVs), averaged per host species. ASVs that were significantly enriched in *C. fluminea* compared to the six native mussels are signaled by an asterisk (*adjusted P<0.05).


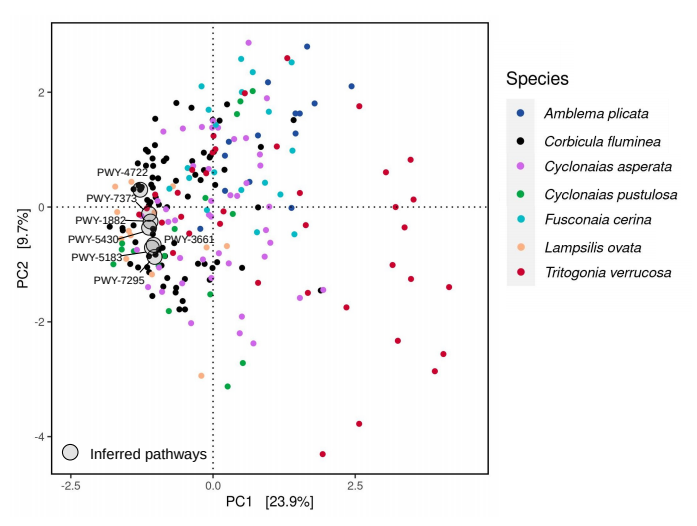
**S2-Figure 4**: Functional inference from 16S rRNA gene data of the gut microbiomes of the invasive species *Corbicula fluminea* and six co-occurring native freshwater mussel species. Specimens were collected in six rivers in the Tennessee and Mobile River Basins. Plots shows a Redondance analysis (RDA) based on the functional pathways inferred in each individual host, with the large circles representing the seven pathways contributing the most to the first two axes of the RDA. Pathways codes are those from the MetaCyc database. PWY-5430: meta cleavage pathway of aromatic compounds; PWY-3661: Glycine betaine degradation I; PWY-7373: Demethylmenaquinone 6 biosynthesis; PWY-1882: Chimeric superpathway of C1 compounds oxydation to CO2; PWY-4722: Creatinine degradation II; PWY-7295: L-arabinose degradation IV; PWY-5183: Superpathway of aerobic toluene degradation.


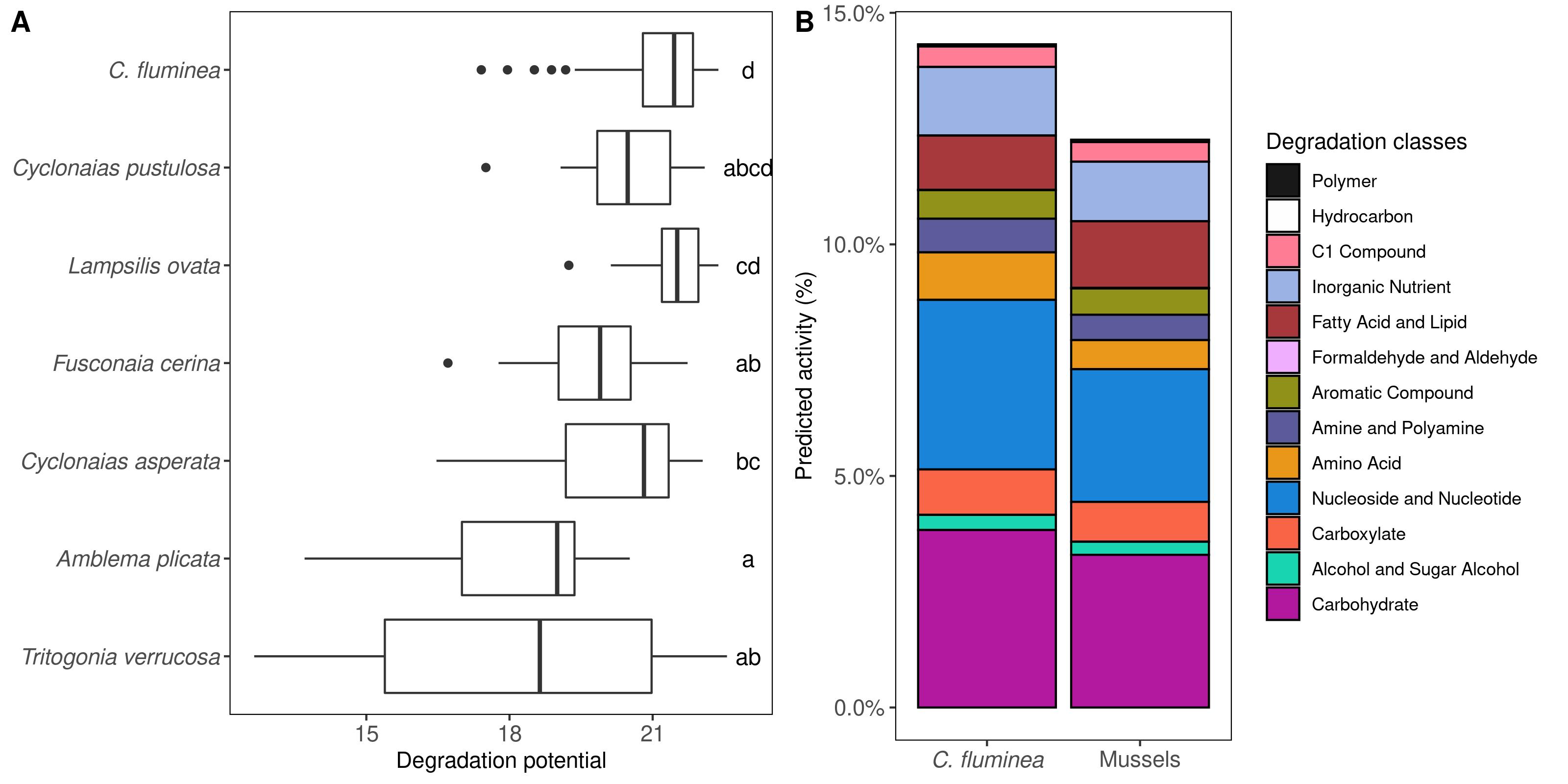


**S2-Figure 5**: Inferred degradation functions of the gut microbiome of the invasive clam *Corbicula fluminea* and six native freshwater mussel species collected from six rivers in the Tennessee and Mobile River Basins. A: Distribution of the degradation potential in all species studied. Comparisons across species were computed using pairwise Wilcoxon tests (P<0.05), which result is indicated by the letters in front of each boxplot. Different letters indicate a significant difference in the distribution of values across the two species considered. B: Aggregated predicted activity of degradation functions per class of compound, averaged for all *C. fluminea* specimens and all mussel species. Classes were recorded from the MetaCyc database and manually regrouped when needed (e.g. alcohols and sugar alcohols).
