# Supplementary material for: Environment and Co-occurring Native Mussel Species, but Not Host Genetics, Impact the Microbiome of a Freshwater Invasive Species (Corbicula fluminea)
Source: Front Microbiol. 2022 Apr 4;13:800061. doi: 10.3389/fmicb.2022.800061 (PMC9014210; doi:10.3389/fmicb.2022.800061)
Supplement: Supplementary file 3 [file Data_Sheet_3.DOCX]

Supplementary ***Information S3: Supplementary Genomics Results***

# **S3-Supplementary Tables**

**S3-Table 1**: Sample sizes, nucleotide diversity, and FIS for the *C. fluminea* RAD-tag dataset.

| **Basin** | **River** | **n** | **π ± SE** | **FIS 95% CI** |
| --- | --- | --- | --- | --- |
| Mobile | Bogue Chitto | 6 | 0.00535 ± 2.76 x 10^-05^ | -0.854 – -0.849 |
|  | Buttahatchee | 10 | 0.00516 ± 2.67 x 10^-05^ | -0.896 – -0.892 |
|  | Sipsey | 41 | 0.00506 ± 2.55 x 10^-05^ | -0.865 – -0.861 |
| Tennessee | Bear Creek* | 1 | NA | NA |
|  | Duck | 101 | 0.00493 ± 2.54 x 10^-05^ | -0.908 – -0.904 |
|  | Paint Rock | 14 | 0.00520 ± 2.63 x 10^-05^ | -0.877 – -0.873 |

**S3-Table 2:** Pairwise F_ST_ estimates for *C. fluminea* from 5,225 SNPs between rivers. All p-values testing for a genetic difference between rivers are significant (P<0.000)

|  | Bogue Chitto | Buttahatchee | Sipsey | Duck | Paint Rock |
| --- | --- | --- | --- | --- | --- |
| Bogue Chitto | - | - | - | - | - |
| Buttahatchee | 0.013 | - | - | - | - |
| Sipsey | 0.011 | 0.003 | - | - | - |
| Duck | 0.015 | 0.001 | 0.003 | - | - |
| Paint Rock | 0.012 | 0.002 | 0.003 | 0.003 | - |

# **S3-Supplementary Figure**


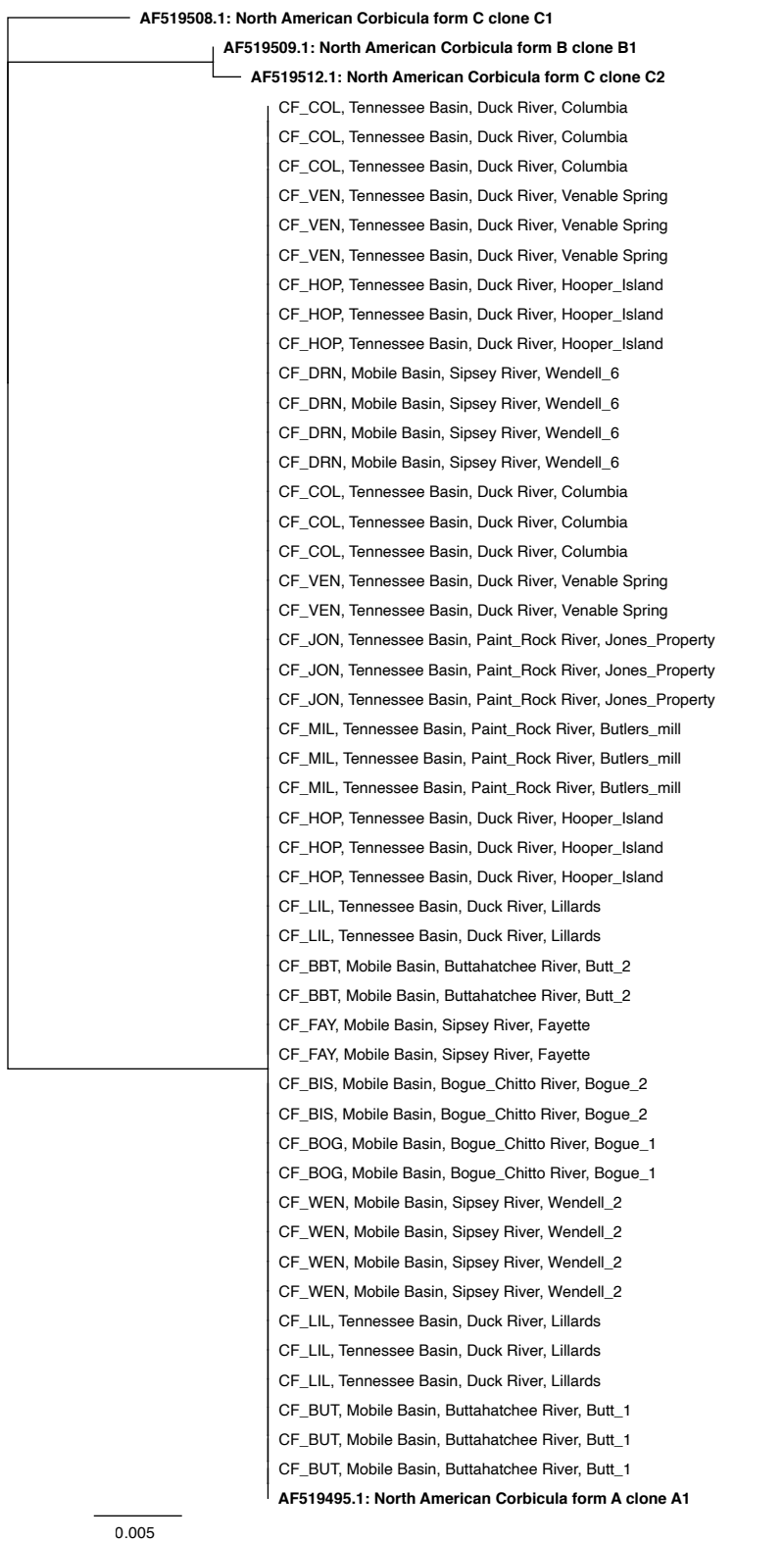


**S3-Figure: Unrooted neighbor-joining tree (constructed in Geneious,** Jukes-Cantor model, other parameters default) of COI sequences (656 bp) from 47 *C. fluminea* specimens sampled from Mobile (Sipsey, Bogue, and Buttahatchee Rivers) and Tennessee (Duck and Paint Rock Rivers) River Basin included in this study. Sequences for different clonal lineages (A, B, C) sampled from GenBank (accession numbers in sequence names) are included for reference and outlined in bold. All sequences for the present study belong to *C. fluminea* lineage A.
